# Before and after study of bar workers' perceptions of the impact of smoke-free workplace legislation in the Republic of Ireland

**DOI:** 10.1186/1471-2458-7-131

**Published:** 2007-06-29

**Authors:** Lisa Pursell, Shane Allwright, Diarmuid O'Donovan, Gillian Paul, Alan Kelly, Bernie J Mullally, Maureen D'Eath

**Affiliations:** 1 Department of Health Promotion, National University of Ireland, Galway, Republic of Ireland; 2 Department of Public Health and Primary Care, University of Dublin, Trinity College, Trinity College Centre for Health Sciences, AMNCH, Tallaght, Dublin 24, Republic of Ireland; 3 Department of Epidemiology and Public Health, University College Cork, Brookfield Health Sciences Complex, Cork, Republic of Ireland

## Abstract

**Background:**

*Objectives*: To compare support for, and perceptions of, the impacts of smoke-free workplace legislation among bar workers in the Republic of Ireland (ROI) pre- and post-implementation, and to identify predictors of support for the legislation.

**Methods:**

*Setting*: Public houses (pubs) in three areas of the ROI.

*Design*: Comparisons pre- and post-implementation of smoke-free workplace legislation.

*Participants*: From a largely non-random selection, 288 bar workers volunteered for the baseline survey; 220 were followed up one year later (76.4%).

*Outcome measures: *Level of support for the legislation, attitude statements concerning potential impacts of the law and modelled predictors of support for the legislation.

**Results:**

Pre-implementation 59.5% of participants supported the legislation, increasing to 76.8% post-implementation. Support increased among smokers by 27.3 percentage points from 39.4% to 66.7% (p < 0.001) and among non-smokers by 12.4% percentage points from 68.8% to 81.2% (p = 0.003).

Pre-legislation three-quarters of participants agreed that the legislation would make bars more comfortable and was needed to protect workers' health. Post-legislation these proportions increased to over 90% (p < 0.001). However, negative perceptions also increased, particularly for perceptions that the legislation has a negative impact on business (from 50.9% to 62.7%, p = 0.008) and that fewer people would visit pubs (41.8% to 62.7%, p < 0.001). After adjusting for relevant covariates, including responses to the attitude statements, support for the ban increased two to three-fold post-implementation. Regardless of their views on the economic impact, most participants agreed, both pre- and post-implementation, that the legislation was needed to protect bar workers' health.

**Conclusion:**

Smoke-free legislation had the support of three-quarters of a large sample of bar workers in the ROI. However, this group holds complex sets of both positive and negative perspectives on the legislation. Of particular importance is that negative economic perceptions did not diminish the widely held perception that the ban is needed to protect workers' health.

## Background

In response to contextual factors including the evidence on the causal link between exposure to secondhand smoke (SHS) and adverse health effects [1,2], in March 2004 the Republic of Ireland (ROI) introduced legislation to effect a ban on smoking in all indoor workplaces, including pubs and restaurants. Although the hospitality sector had been highlighted as an environment in which workers have consistently high exposure to SHS [1,3-5] prior to implementation many concerns were expressed about the consequences of comprehensive smoke-free workplace legislation on the public bar trade in the ROI [6,7]. These concerns were mainly fears about the loss of jobs and trade in the hospitality industry.

Previous studies have examined perceptions concerning the impacts of smoke-free legislation within the hospitality industry and among patrons. Restaurant owners' pre-legislation perceptions of the business prospects have been reported by Hammer [8] in Gothenburg, Sweden and Crémieux [9] in Quebec, Canada and support for smoke-free legislation among restaurateurs in Adelaide, Australia was examined by Jones *et al*. [10]. A UK based study examined the attitudes of London casino workers to SHS and found the majority of respondents wanted all working areas in their casino to be smoke-free [11]. Tang and colleagues [12] reported increasing support and compliance with smoke-free legislation among bar patrons after implementation in California [12]. Fong and colleagues [13] found that among smokers in Ireland support for a total ban on smoking in pubs increased by 13% to 46% eight to nine months after implementation of the smoke-free legislation.

However, there are few studies of attitudes to smoke-free legislation that focus on bar workers. In a study of bar staff and owners in California, Tang and colleagues [14] found significant positive changes in attitudes to a smoke-free law shortly after its enactment and four years later. Milne and Guenole [15] assessed attitudes and beliefs of bar workers about the extension of the New Zealand workplace smoke-free law to hospitality venues before and after the legislation was introduced in December 2004. The All Ireland Bar Study was set up to assess the impact of the Republic's smoke-free legislation on exposure to SHS, on respiratory health, on attitudes to the legislation and on smoking rates of bar workers. Allwright and colleagues [16] have reported the effects of the legislation on SHS exposure and on respiratory symptoms. The present study presents an assessment of the impact of the legislation on attitudes of bar workers towards the legislation.

We examined support for the smoke-free workplace legislation among bar workers and compared their perceptions of the impacts of the legislation before and after implementation of the law. We examined these perceptions and other participant characteristics as predictors of support for the smoke-free legislation (smoking ban) before and after the law was implemented.

## Methods

Bar workers from public houses (pubs) in three areas of the ROI (Dublin city, Cork city and Galway county) were enrolled in a baseline questionnaire survey between September 2003 and March 2004. The three areas were selected to represent different types of pub environment. In light of the tensions regarding the legislation at this time, sampling procedures were adapted to local circumstances to maximise follow-up. Only the Cork sample was randomly selected. In Dublin, we selected city centre and suburban pubs. The main trade union for Dublin bar workers (Mandate) forwarded our letter asking members to contact the research team if they were interested in participating. In County Galway all pubs located in electoral divisions with populations of less than 1500 were invited by letter to participate in the survey. All staff present on the day of the survey were asked to participate. In Cork city all pubs on randomly selected streets were invited to participate in the study and up to two bar staff were randomly selected from each pub. A follow-up survey post-legislation was completed one year later. (See Appendix for more details on the Galway and Cork sampling.)

### Demographics

At both baseline and follow-up, participants were asked about age, gender, self reported smoking status, occupational sub-group, type of pub, relationship to the pub owner, number of hours worked per week on average and number of years worked in bars.

### Assessment of support for the smoke-free legislation

Overall support for the legislation was determined by asking the question, "Do you agree with the ban on smoking?" Perceptions of the smoke-free legislation were assessed with a series of statements derived from issues highlighted during the period between announcement of the legislation and its implementation. The statements were derived from several sources. Perceived positive and negative aspects of the legislation were identified as part of a study on the feasibility of a Health Impact Assessment based on methods described by Ison [17]. The main theme areas were identified from documentary analysis of written submissions to the public consultation process, from transcripts of the public meetings organised by the Health and Safety Authority [18] and from media analysis of the major print media for a 12 month period before the legislation. The majority of views expressed during that period concerned the loss of jobs and the potential for a downturn in trade in the hospitality industry.

The statements were presented at baseline and follow-up and participants were asked to respond to each statement on a five-point scale, ranging from 'strongly agree' to 'strongly disagree' (midpoint: 'undecided'). Prior to analysis responses were aggregated to create dichotomous variables. For positive statements, 'strongly agree' and 'agree' were combined to form the category 'agree'; 'undecided', 'disagree' and 'strongly disagree' were categorised 'not agree'. To test for active disagreement with negative statements 'strongly disagree' and 'disagree' were aggregated to form the category 'disagree'; the other responses were categorised 'not disagree'. The same five point scale was used for responses to the overall support question "Do you agree with the ban on smoking?"

### Data handling and definitions

*Age*- Age was categorised into two groups, less than or equal to the median age in years, and greater than the median age.

#### Smoking status

Participants were asked to report their smoking status as: regular smoker, occasional smoker, ex-smoker or never smoked. Salivary cotinine levels were measured in the baseline and follow-up surveys by the technique used by the Health Survey for England [19] in order to assess levels of SHS exposure and to validate the self-reported smoking status. Ex-smokers and never-smokers with cotinine levels <20 ng/ml (113.6 nmol/l) were defined as non-smokers [16].

#### Occupational position

Two variables contributed to the definition of occupational position: relationship to the bar owner and self-reported occupational status within the pub trade. Pub owners, leaseholders or staff reporting a relationship to the owner as son, wife, daughter, in-law or friend, were combined in the category 'owner'. Respondents defined as 'employee' included those who described themselves as manager, permanent bar staff, or temporary bar staff and included those who reported a relationship with the pub owner as employee.

#### Pub location

Participants were categorised as rural or urban bar workers according to the population size surrounding the pub's location. Pubs were defined as rural if located in an electoral division with a population < 1500, while urban pubs were defined as those in locations with electoral division populations ≥ 1500 [20].

#### Family-run pub

Participants were categorised as working in a family-run pub based on their response to a question asking if the pub they owned or worked in was a family-run pub.

### Statistical analysis

Comparability of those followed up and those not followed up was tested using Pearson's X^2 ^for categorical variables and the Wilcoxon rank sum test for medians. Differences between categorical variables (e.g. occupational position, pub location, smoking status) at both baseline and follow-up were tested using Pearson's X^2^. Analyses relate to changes in perceptions of the legislation (agreement/disagreement with the ban or with statements posing positive or negative perspectives on the ban) between baseline and follow-up. Analyses of within pair changes to attitudes between baseline and follow-up were restricted to individuals who took part in both baseline and follow-up surveys and who were still working in a pub. For characteristics that may have changed over time (e.g. smoking status, occupational position, pub location) internal control was maintained by restricting analyses to those who did not change status for these variables between baseline and follow-up (tested using Pearson's X^2^). McNemar's X^2 ^or exact test was used to test for significant changes between baseline and follow-up in paired categorical variables (i.e. agree or not agree).

To model predictors of support for the legislation and change over time a logistic regression version of generalised estimation equations (GEE) was used to give odds ratios (Stata 9, StataCorp, College Station, TX). All covariates were tested in the model but only those that achieved or approached significance (p < 0.10) were retained. In order to orientate all responses in the models with respect to support for the legislation, responses to positive statements were presented as proportions who agreed and strongly agreed, and responses to negative statements were presented as proportions who disagreed and strongly disagreed.

To test for the effect of negative economic perspectives on perceptions of health benefits and overall support for the legislation, responses to the statement expressing the need to protect workers' health was stratified by responses to perspectives on negative economic impacts and by overall support for the legislation. Stratified responses were analysed using Pearson's X^2^.

## Results

In total 207 bar workers were enrolled at baseline from 139 pubs in Galway and Cork, and a further 81bar workers were enrolled in Dublin from Mandate Trade Union. Of the 288 bar workers enrolled in the baseline study in the ROI, 220 were enrolled for the follow-up survey (76.4% overall and 88.7% of those eligible for follow-up; see Appendix for details of County Galway and Cork city pub recruitment).

Participants who were followed up were similar to those surveyed only at baseline with respect to number of hours worked, occupational position, pub location and support for the legislation (Table 1). However, those followed up tended to be older, more likely to be male, non-smokers, work in family-run pubs and have worked for longer in the current pub.

**Table 1:** Baseline characteristics of bar staff followed up (n = 220) and not followed up (n = 68)

	**Followed up**	**Not followed up**	p*
Age in years, median (IQR)	42 (28–52.8)	26 (21–33)	<0.001
Gender (female) n (%)	48 (21.8)	26 (38.2)	0.006
Length of time worked in bars, median years (IQR)	15.5 (3–16)	7 (3–12)	<0.001
Average number of hours per week worked, median (IQR)	40 (39–50)	40 (38–50)	0.182
Work in a family-run pub, n (%)	138 (63.9)	30 (44.1)	0.003
Smoking status† (current smoker) n (%)	79 (35.9)	44 (64.7)	<0.001
Occupational position‡ (owners), n (%)	75 (34.7)	20 (29.9)	0.280
Pub location§(rural) n (%)	48 (22.5)	20 (29.4)	0.161
Support the ban (agree¶) n (%)	131 (59.5)	35 (51.5)	0.239

IQR: Interquartile range.

* p value for Wilcoxon rank sum test for comparison of medians and Pearson's χ^2 ^for categorical variables.

† Smoking status categorised by current or non-smoker (current smokers defined as self reported or occasional smokers plus self reported never or ex smokers with salivary cotinine levels ≥ 20 ng ml^-1^.

‡ Occupational position categorised as employee (manager or permanent or temporary bar staff) or owner (pub owners, leaseholders, or owner's relatives or friends).

§Pub location categorised by rural or urban location of the pub (rural pubs defined as pubs located where the population was <1500 and urban pubs as those where the population was ≥ 1500).

¶Combines sub-groups 'strongly agree' and 'agree'

### Support for the legislation

The proportions agreeing with the legislation were compared for various sub-groups and the two time periods (Table 2). Significant differences (p < 0.05) between the sub-groups at baseline were recorded for all variables, with the exception of pub location and working in a family-run pub. Support for the legislation was more likely among bar workers who were male, over 42 years, non-smokers, employees and who worked shorter hours per week. With the exception of those aged over 42 years, support increased significantly (p < 0.05) in all sub-groups at follow-up, particularly among those working over 40 hours per week, owners and smokers.

**Table 2:** Support for the smoke-free workplace legislation (strongly agree and agree) by various characteristics of participants, n (*%*) at baseline and follow-up

		**Baseline support**		**Follow-up support**		**Increase in support**	
		
		n (*%*)	p*	n (*%*)	P*	n (percentage points)	p†
Overall		131 (59.5)		169 (76.8)		38 (17.3)	<0.001
Age (n = 219)§/P >	≤ 42 years (n = 117)	57 (48.7)	0.001	88 (75.2)	0.632	31 (18.2)	<0.001
	> 42 years (n = 102)	73 (71.6)		80 (78.4)		7 (5.4)	0.21
Gender (n = 220)	female (n = 48)	19 (39.6)	0.001	29 (60.4)	0.002	10 (20.8)	0.021
	male (n = 172)	112 (65.1)		140 (81.4)		28 (16.3)	<0.001
Smoking status¶(n = 204)	current smoker (n = 66)	26 (39.4)	<0.001	44 (66.7)	0.022	18 (27.3)	<0.001
	non-smoker (n = 138)	95 (68.8)		112 (81.2)		17 (12.4)	0.003
Occupational status** (n = 216)	owner (n = 75)	31 (41.3)	<0.001	48 (64)	0.002	17 (22.7)	0.004
	employee (n = 141)	96 (68.1)		117 (83)		21 (14.9)	<0.001
Pub location†† (n = 213)	rural (n = 48)	23 (47.9)	0.085	33 (68.8)	0.123	10 (20.9)	0.031
	urban (n = 165)	102 (61.8)		131 (79.4)		29 (17.6)	<0.001
Hours worked per week§(n = 169)	≤ 40 hours (n = 97)	67 (69.1)	<0.001	76 (78.4)	<0.001	9 (9.3)	0.049
	> 40 hours (n = 72)	25 (34.7)		51 (70.8)		26 (36.1)	<0.001
Work in family-run pub (n = 216)	yes (n = 138)	75 (54.3)	0.051	99 (71.7)	0.018	24 (17.4)	<0.001
	no (n = 78)	53 (67.9)		67 (85.9)		14 (18.0)	0.001

Analysis included only those surveyed at both baseline and follow-up. Respondents who reported a change in status (e.g. occupational position, pub location, smoking status) between surveys were excluded from the analysis.

* p value for Pearson's χ^2 ^comparing support for the legislation by sub-groups.

† p value for McNemar's χ^2 ^comparing changes in support for the legislation at baseline and follow-up.

§Continuous variable dichotomised around the median.

¶Smoking status categorised by current or non-smoker (current smokers defined as self reported or occasional smokers plus self reported never or ex smokers with salivary cotinine levels ≥ 20 ng ml^-1^).

** Occupational position categorised as employee (manager or permanent or temporary bar staff) or owner (pub owner, leaseholder, or owner's relative or friend).

††Pub location categorised by rural or urban location of the pub (rural pubs defined as pubs located where the population was <1500 and urban pubs as those where the population was ≥ 1500).

### Perceived impacts of the legislation

Perceptions of the smoke-free legislation were assessed with the series of statements presented in Table 3. The modifications used in the follow-up survey are shown in parentheses. Before the legislation three-quarters of the participants agreed that the ban would make bars more comfortable and was needed to protect workers' health (Table 3). Post-legislation the proportion increased to over 90% (p < 0.001). There was a non-significant decrease in those perceiving that the ban would encourage smokers to quit. With respect to the five negative statements, at follow-up there was an increase in the proportions agreeing with four of these statements but the increase was significant for only two of the statements (that fewer people would visit bars and that the smoking ban would make smokers smoke more at home). However, there was a slight decrease in those who thought that the smoking ban was an unfair restriction on smokers (Table 3).

**Table 3:** Perceptions of positive impacts of the smoke-free workplace legislation (strongly agree and agree for positive statements; strongly disagree and disagree for negative statements) at baseline and follow-up (n = 220) and impact of these attitudes on support for the legislation and changes in support after implementation

			**CHANGES AT FOLLOW-UP**			**SUPPORT FOR THE BAN**	
	**BASELINE**	**FOLLOW-UP**	**No change**	Net change*	***p ***†	**Adjusted OR by statement **‡ (CI)	**Adjusted OR by time period **§(CI)

	n (%)	n (%)	n (%)	n (*percentage points*)			
**Positive statements**	**Strongly agree and agree**						

Smoke-free bars will be (are) more comfortable to visit	170 (77.6) ¶	199 (90.5)	181(82.3)	28(12.8)	<0.001	29.86 (11.26 to 79.19)	2.07 (1.23 to 3.46)
The smoking ban is needed to protect the health of workers	180 (81.8)	201 (91.8) ¶	183 (83.2)	22 (10)	<0.001	15.24 (5.99 to 38.82)	2.11 (1.32 to 3.37)
The smoking ban will encourage (has encouraged) smokers to quit	155 (72.4) **	147 (67.1) ¶	152 (69.1)	-13 (-5.5)	0.124	4.06 (2.39 to 6.89)	3.27 (2.07 to 5.15)

**Negative statements**	**Strongly disagree and disagree**						

The ban on smoking will have (has had) a negative effect on business	62 (28.2)	56 (25.5)	150 (68.2)	-6 (-2.8)	0.550	3.71 (1.98 to 6.98)	2.66 (1.73 to 4.10)
Fewer people will visit (have visited) bars after (since) the ban	90 (40.9)	57 (25.9)	135 (61.4)	-33 (-15)	0.001	4.10 (2.29 to 7.35)	3.26 (2.06 to 5.16)
The smoking ban is an unfair restriction on smokers	109 (50.2) ††	123 (55.9)	170 (77.3)	13 (6.1)	0.080	18.89 (9.67 to 36.90)	3.14 (1.84 to 5.36)
The smoking ban will make (has made) smokers smoke more at home	87 (39.9) ‡‡	51 (23.2)	143 (65)	-37 (-16.8)	<0.001	1.72 (1.01 to 2.92)	2.91 (1.88 to 4.50)
The smoking ban will result (has resulted) in jobs being lost	81 (36.8)	78 (35.5)	153 (69.5)	-3 (-1.4)	0.807	4.19 (2.34 to 7.50)	2.84 (1.81 to 4.45)

* Calculated as the net change in agreement with the statement (the percentage increase in agreement with the statement minus the percentage decrease in agreement).

† p value for McNemar's χ^2 ^comparing changes in agreement with the statements at baseline and follow-up.

‡ Odds ratio for support for the ban by response to each statement adjusted for change over time (follow-up survey:baseline survey), age, gender (male:female), occupational position (owner:employee) and smoking status (current smoker:non-smoker).

§Odds ratio for support for the ban at follow-up relative to baseline adjusted for potential change in response to statement, age, gender (male:female), occupational position (owner:employee) and smoking status (current smoker:non-smoker).

¶1 respondent did not answer this question.

** 6 respondents did not answer this question.

†† 3 respondents did not answer this question.

‡‡ 2 respondents did not answer this question.

The penultimate column in Table 3 presents the odds of support for the ban according to agreement (or disagreement) with each statement adjusting for changes over time (follow-up relative to baseline) and other relevant covariates. For example, the odds of agreeing with the legislation were 30 greater (adjusted OR = 29.86, CI: 11.3 to 80) among those who agreed that the ban would make smoke-free bars more comfortable than among those who did not agree that the ban would make bars more comfortable. The odds of supporting the ban were four times (CI: 1.98 to 6.98) greater among those who did not agree with the statement, "The ban will have (has had) a negative effect on business"; and 19 times (CI: 9.67 to 36.9) greater among those who did not agree with the statement, "The ban is an unfair restriction on smokers".

The last column in Table 3 presents the odds of support for the ban at follow-up relative to baseline, adjusted for potential change in agreement (or disagreement) with each statement in turn and other relevant covariates: the adjusted odds of supporting the legislation increased two to three-fold post-implementation.

Smokers were between 20% and 50% less likely to support the legislation. (The adjusted odds ratios for smoker:non-smoker ranged between 0.49 and 0.80 for the various attitude statements; data not shown in table.)

### Health concerns vs. economic concerns

Among the positive statements listed in Table 3, the highest level of agreement at both baseline and follow-up was for the need to protect workers' health. On the other hand, only about a quarter of the participants (28.2% at baseline and 25.5% at follow-up) did not agree with the statement "The ban on smoking will have (has had) a negative effect on business" (i.e. most thought that the legislation would have a negative effect on business).

In Table 4, support for the smoke-free law in general has been stratified by agreement with the legislation being needed to protect workers' health and by perceptions of a negative impact on business. Stratification revealed that among participants who supported the legislation, over 90% thought that the ban was needed to protect workers' health, irrespective of whether they agreed that it would have a negative effect on business (p = 0.3). Approximately 60% of those who did not support the legislation in general nevertheless agreed that it was needed to protect workers' health and this response was similarly unaffected by concerns about negative economic consequences (p = 0.8). This pattern held for both baseline and follow-up, but with increased agreement at follow-up with the statement on protecting workers' health.

**Table 4:** Responses to the statement expressing the need to protect workers' health stratified by overall support for the smokefree workplace legislation and by responses to perspectives on negative economic impacts

**Do you agree with the proposed ban on smoking**	**The ban on smoking will have a negative effect on business**	**The smoking ban is needed to protect the health of workers**			
		**Baseline n (%)**		**Follow-up n (%)**	
		
		**Agree**	P*	**Agree**	P*
Agree†	Agree (n= b 52; f 92) ‡	49 (94.2)	0.300	90 (97.8)	1.000
	Not agree (n= b 79; f 76)	78 (98.7)		75 (98.7)	
	Total (n= b 131; f 168)	127 (96.9)		165 (98.2)	

Not agree§	Agree (n= b 60; f 45)	35 (58.3)	0.820	32 (71.1)	1.000
	Not agree (n= b 29; f 6)	18 (62.1)		4 (66.7)	
	Total (n= b 89; f 51)	53 (59.6)		36 (70.6)	

*P value for Pearson's χ^2 ^comparing responses to the statement that the ban is needed to protect workers' health by support for the legislation by responses to the statement that the ban will have a negative effect on business.

† Agree = strongly agree and agree

‡ b = baseline; f = follow-up

§Not agree = strongly disagree, disagree and undecided

## Discussion

### Main findings

This study shows that, contrary to expectations, opposition to the smoke-free legislation among bar workers was not widespread and that experience of working in a smoke-free environment led to increased support. The largest increases in support occurred among smokers, pub owners and rural pub staff, groups who had been highlighted in the media as being particularly opposed to the legislation pre-implementation. This result is consistent with the increased support among smokers in the ROI reported by Fong *et al*. [13] and with the findings from a survey of bar managers' attitudes in New Zealand [15].

#### Positive perceptions

The majority of participants believed that the legislation was needed to protect workers' health irrespective of whether or not they supported the legislation and this perception increased post-implementation. Table 4 shows that agreement with the statement that bar workers' health should be protected was unaffected by concerns about negative economic impacts. Participants also initially appreciated the potential for quitting smoking. However, post-implementation they perceived the reality as less impressive than anticipated. An apparent partial reversal of the post-legislation decline in smoking prevalence in the ROI nine months later may support this view but future trends remain to be confirmed [21]. This is in contrast to the International Tobacco Control (ITC) survey which reported that 80% of those who had quit since the legislation thought that it had helped them do so [13].

#### Negative perceptions

Participants had initially been concerned about the unfairness of the legislation to smokers, but were less concerned post-implementation. Their key worry pre-implementation was about economic impacts and this concern increased post-implementation. This is hardly surprising given the tobacco industry's worldwide propaganda campaign around the "myth of lost profits" and the "accommodation" of smokers [22].

The Central Statistics Office's retail volumeindices for the Irish pub trade started to decline for the first time in 2001, threeyears before the smoke-free law was introduced [23]. This decline has been attributed to a number of factors such as increased vigilance towards underage drinking, increasing prices in pubs and changing social patterns in alcohol consumption, including an increasing awareness of the need to avoid drink driving. There was nosignificant change in theannualrate of decline following the introduction of thelegislation(4.2% decline in 2003, 4.4% in 2004). By mid 2005 sales started to increase for the first time in four years, (December 2005 returns reported annual percentage increases in the value and volume indices of 1.3% and 0.1% respectively and preliminary estimates of annual percentage increases for the year ending December 2006 of 2.5% and 0.9% respectively [23]), thus gainsaying the doomsday scenario predicted by the hospitality industry. These trends are consistent with the trends recorded in other countries such as New Zealand, USA, Norway and Canada [9,24-26]. It is now generally accepted among the hospitality trade that the smoke-free law was only one of several factors affecting the current trend away from pub sales to off license sales [27].

Post-legislation a small rise in the numbers concerned about increased home smoking was recorded, although this perception is not supported by the findings of Allwright *et al*., which revealed that self-reported exposure to SHS outside of work had dropped significantly in the ROI [16]. Fong *et al*., also reported a significant decrease in the percentage of Irish homes where smoking was allowed [13]. Waa and McGough [28] reported that self-reported exposure to SHS in homes decreased after the smoke-free legislation was extended to cover hospitality venues in New Zealand.

Table 4 shows that the belief that bar workers' health should be protected was unaffected by their concerns about negative economic impacts. This suggests that promoting a strong health protection message may have greater impact on the level of support for legislation than anxieties about possible negative economic impacts.

### Comparison with other studies

Few follow-up studies of bar workers' attitudes to smoke-free legislation have been conducted. A survey of bar managers' attitudes in New Zealand showed that overall support for the smoking legislation increased significantly after implementation, but, contrary to our findings, agreement that smoke-free laws do not affect patron numbers and venue profits also increased [26]. A study in California focusing on bar workers compared attitudes to smoke-free legislation shortly after implementation and four years later [14]. Although not initially as high as in the present study the proportion having positive attitudes to the legislation increased significantly after four years, particularly with respect to concerns about the health effects of second-hand smoke and for the preference to work in smoke-free environments. In this respect it would be interesting to see if similar increases pertain among Irish bar workers four years on.

An earlier study in New Zealand of bar and restaurant workers' perceptions and attitudes to SHS did not use a follow-up design and focused on one location [29]. Other studies of attitudes to smoke-free legislation in Indiana [30] and California [12,14,31] were based on general population samples. A Norwegian study focused on hospitality workers using a follow-up design but did not differentiate between bar and restaurant workers [32].

### Study strengths

Our multi-centre study provided a sample of bar workers from both rural and urban areas across the Republic of Ireland. The study used a longitudinal design enabling within-pair, before and after comparisons. The focus of this study was a group of workers who had been considered to be highly vulnerable to potential negative economic impacts. While concerns persisted around this issue post-legislation, the study revealed a significant increase in general support for the legislation. This outcome was further reinforced by their reported perceptions of health benefits outweighing perceptions of negative economic impacts.

### Study limitations

Most of the study sample comprised volunteers and as such, the proportions reporting support for the legislation may not be generalisable. However, the sample in one area (Cork) was selected randomly and differences in support for the legislation between the random sample (Cork) and the total sample (ROI), when compared by smoking and occupation sub-groups, were non-significant (to be reported elsewhere). A number of baseline participants were lost to follow-up. However, they showed similar levels of support for the legislation to those followed up. Participants may have responded to the impact statements in a manner that they thought we would want to hear. However, complex sets of positive and negative perspectives on the various impacts were recorded that in the majority of cases co-existed with overall support for smoke-free legislation.

### Policy implications

Since the smoking legislation was introduced in the Republic, further studies have provided evidence on the health benefits of smoke-free policies such as reduced respiratory symptoms [16,33] and reduction in hospital admissions for myocardial infarction [34-36].

Our study has shown that, contrary to expectations, opposition to the smoking legislation among bar workers was not widespread and that experience of working in a smoke-free environment led to increased support, especially among smokers and bar owners and managers. There was concern pre-legislation about adverse economic impacts and this persisted post-legislation, in spite of media reports presenting evidence that there was no additional economic decline. This suggests that there is a need to make greater efforts to provide the hospitality sector with factual local and international information to counteract the tobacco industry's efforts to promote "the myth of lost profits" [22]. In light of the finding that negative economic impacts are more likely in studies using subjective outcome measures [37], an analysis of the economic impact in Ireland using objective data would be helpful. However, the fact that concerns about perceived negative economic impacts did not affect participants' belief that bar workers' health should be protected, suggests that promoting a strong health protection message may have a greater impact on increasing support for legislation than perceptions of negative economic impacts. This is of interest in light of the decision to make the protection of worker health the primary focus of the Irish smoke-free campaign.

### Recommendations for further research

This study has shown that while the smoke-free legislation has been perceived as generally beneficial by the majority of a large sample of Irish bar workers, issues regarding economic impacts and the possibility of increased smoking in the home were still of concern six to 12 months later. It would be interesting to repeat the survey several years after the legislation to see if bar workers continue to have these concerns. Other jurisdictions considering smoke-free legislation may wish to consider prioritising investigation of these issues using valid longitudinal approaches. A thorough analysis, employing objective outcome measures, of the economic impact of the legislation in the ROI is required.

### What this study adds

There have been few previous studies of attitudes pre- and post smoke-free legislation that focus exclusively on bar workers. After introduction of the legislation in the Republic of Ireland support for the legislation among bar workers increased significantly, particularly among smokers and owners. Perceptions of health benefits outweighed concerns about possible negative economic impacts.

## Conclusion

Contrary to expectations, opposition to the smoking legislation among bar workers was not widespread and experience of working in a smoke-free environment led to increased support, especially among smokers and bar owners and managers. Pre-legislation concern about adverse economic impacts persisted post-legislation. However, concerns about perceived negative economic impacts did not diminish the widely held perception that the ban is needed to protect workers' health.

## Competing interests

SA is a member of the Board of the Irish Office of Tobacco Control (unpaid position).

## Authors' contributions

Contributors: All authors reviewed and approved the final version of this manuscript.

LP participated in research design, preparation of study materials and protocol, performed most of the analyses, wrote all drafts of the paper, and is guarantor.

SA conceptualised the study and was involved in and coordinated all aspects of the study, contributed to preparation of study materials, interpretation of findings, and co-wrote the drafts.

DO'D participated in the research design and protocol preparation, contributed to preparation of study materials, interpretation and commented on drafts.

GP participated in design, preparation of study materials and protocol, data collection, data entry/editing, interpretation, and commented on drafts.

AK was statistical adviser, conducted statistical modelling, and participated in the interpretation.

BJM participated in design, preparation of study materials and protocol data collection, data entry/editing, and commented on drafts.

MD'E participated in data collection, data entry/editing, and commented on drafts.

**Ethical approval:**Research ethics committee of the Faculty of Public Health Medicine, Royal College of Physicians of Ireland; the St. James's Hospital and Federated Dublin Voluntary Hospitals joint research ethics committee; the clinical research ethics committee of the Cork Teaching Hospitals; and the healthcare committee and senior management team of the Western Health and Social Services Board and the Western Investing for Health Partnership.

## Appendix

### Galway pub selection

The Galway pubs were identified (July 2003) from the web-based Golden Pages listings [38]. Search criteria were: public houses or lounge pubs in County Galway. 344 premises were identified using these criteria. From this total all premises located in electoral divisions with populations at or above 1500 were excluded, leaving 179 premises. Introductory letters were sent to each of the remaining premises, inviting participation in the study. Follow-up telephone calls were made to each address contacted. The owners or managers of 48 premises agreed to participate in the study.

At follow-up 41 pubs participated in the study: 7 pubs with 2 bar workers per pub and 34 with 1 bar worker per pub (n = 48).

### Cork pub selection

In Cork city all pubs on randomly selected streets were invited to participate in the study. Of the 171 pubs identified on these streets, 98 pubs participated, 30 were closed, 8 pubs refused and 35 could not be surveyed due to time constraints. A random selection of up to 2 bar workers present at the time of visit was interviewed in each pub. (All were invited to participate if there were 2 or fewer bar workers present). If a randomly selected bar worker was unable or unwilling to participate a replacement bar worker was then randomly selected, if possible, from the same pub (9%).

At follow-up 98 pubs participated in the study: 31 pubs with 2 bar workers per pub and 67 with 1 bar worker per pub (n = 129).

## Funding

Office of Tobacco Control through the Research Institute for a Tobacco Free Society (Republic of Ireland); the National Cancer Institute of the United States (R01 CA90955); Irish Cancer Society; Irish Heart Foundation, Health Service Executive, Western Area, Ireland and the Department of Epidemiology and Public Health, University College Cork, Ireland. Mandate Trade Union provided two prizes for a draw.

## Pre-publication history

The pre-publication history for this paper can be accessed here:


